# Functional Analysis of the Rice Type-B Response Regulator RR22

**DOI:** 10.3389/fpls.2020.577676

**Published:** 2020-11-10

**Authors:** Maria V. Yamburenko, Jennifer M. Worthen, Asyia Zeenat, Beenish J. Azhar, Swadhin Swain, Adam R. Couitt, Samina N. Shakeel, Joseph J. Kieber, G. Eric Schaller

**Affiliations:** ^1^Department of Biological Sciences, Dartmouth College, Hanover, NH, United States; ^2^Department of Biochemistry, Quaid-i-Azam University, Islamabad, Pakistan; ^3^Department of Biology, University of North Carolina, Chapel Hill, NC, United States

**Keywords:** type-B response regulator, cytokinin, rice, panicle, grain yield, trichome, root architecture, silica cell

## Abstract

The phytohormone cytokinin plays a critical role in regulating growth and development throughout the life cycle of the plant. The primary transcriptional response to cytokinin is mediated by the action of the type-B response regulators (RRs), with much of our understanding for their functional roles being derived from studies in the dicot Arabidopsis. To examine the roles played by type-B RRs in a monocot, we employed gain-of-function and loss-of-function mutations to characterize *RR22* function in rice. Ectopic overexpression of *RR22* in rice results in an enhanced cytokinin response based on molecular and physiological assays. Phenotypes associated with enhanced activity of *RR22* include effects on leaf and root growth, inflorescence architecture, and trichome formation. Analysis of four *Tos17* insertion alleles of *RR22* revealed effects on inflorescence architecture, trichomes, and development of the stigma brush involved in pollen capture. Both loss‐ and gain-of-function *RR22* alleles affected the number of leaf silica-cell files, which provide mechanical stability and improve resistance to pathogens. Taken together, these results indicate that a delicate balance of cytokinin transcriptional activity is necessary for optimal growth and development in rice.

## Introduction

The phytohormone cytokinin plays a critical role in regulating growth and development throughout the life cycle of the plant, including the regulation of cell proliferation, shoot and root architecture, seed yield, senescence, and stress responses (Sakakibara, 2006; Hwang et al., 2012; Kieber and Schaller, 2014; Jameson and Song, 2016). Much of our understanding of the mechanisms that underlie metabolism and perception of cytokinin come from studies of the model dicot Arabidopsis (Hwang et al., 2012; Kieber and Schaller, 2014). More recently, such studies have been extended to monocots due to their agronomic importance, with rice being a preferred monocot species for such studies in part because of its small genome and ease of transformation (Devos and Gale, 2000). Of particular interest has been the discovery that cytokinin plays a key role in regulating the development and architecture of the rice inflorescence, alterations in cytokinin levels accounting for differences in the yield for several rice varieties (Ashikari et al., 2005; Kurakawa et al., 2007; Gu et al., 2015; Yeh et al., 2015).

The cytokinin signal is transduced through a multi-step phosphorelay that incorporates cytokinin receptors with histidine-kinase (HK) activity, histidine-containing phosphotransfer proteins (AHPs), and type-B response regulators (RRs; Ito and Kurata, 2006; Pareek et al., 2006; Du et al., 2007; Schaller et al., 2007; Werner and Schmülling, 2009; Tsai et al., 2012; Kieber and Schaller, 2014). Activation of the receptors initiates the transfer of phosphate from one signaling element to the next, thereby relaying the cytokinin signal from membrane to nucleus. Within the nucleus, the type-B RRs function as transcription factors to regulate the expression of cytokinin primary-response genes. Among the primary-response genes are a second family of RRs, the type-A RRs, which function as negative feedback regulators to desensitize the plant to cytokinin (To et al., 2004; Kieber and Schaller, 2014). The KMD family of F-box proteins targets the type-B RRs for degradation (Kim et al., 2013a,b). In rice, as in most plants, these signaling elements are encoded by multi-gene families, there being 13-type-B *RR*s and 13 type-A *RR*s in the rice genome (Pareek et al., 2006; Du et al., 2007; Schaller et al., 2007; Tsai et al., 2012).

According to this model for the cytokinin signal transduction pathway, the type-B RRs play a key role in regulating the initial transcriptional response to cytokinin. The type-B RRs contain two conserved signaling motifs: a receiver domain that is phosphorylated on a conserved aspartate residue to regulate their activity, and a long C-terminal extension with a Myb-like DNA-binding domain (Imamura et al., 1999; Hosoda et al., 2002). Multiple lines of evidence support the role of type-B RRs as transcription factors (Sakai et al., 2000, 2001; Hwang and Sheen, 2001; Imamura et al., 2001, 2003; Lohrmann et al., 2001; Hosoda et al., 2002; Mason et al., 2004, 2005; Rashotte et al., 2006; Liang et al., 2012; Tsai et al., 2012), and our recent analyses using protein-binding microarrays reveal similar DNA-binding motifs for the Myb-like domains of the type-B RRs from rice and Arabidopsis (Raines et al., 2016; Zubo et al., 2017). The type-B RRs fall into five subfamilies based on phylogenetic analysis of rice and Arabidopsis (Tsai et al., 2012), with subfamily-1 being the only subfamily to contain type-B RR members from both rice and Arabidopsis. Genetic analysis in Arabidopsis indicates that subfamily-1 plays the most prominent role in cytokinin signaling (Mason et al., 2005; Yokoyama et al., 2007; Argyros et al., 2008; Ishida et al., 2008).

We have employed several approaches to functionally characterize the role of the rice type-B *RR*s. Initially, we determined that the rice *RR22* of subfamily-1 can complement an Arabidopsis type-B *RR* mutant (*arr1/12*), consistent with rice subfamily-1 also mediating cytokinin responses (Tsai et al., 2012). More recently, we employed a CRISPR/Cas9 gene editing approach to target the four most abundant type-B *RR*s of rice subfamily-1: *RR21*, *RR22*, *RR23*, and *RR24*. Results from this analysis revealed functional overlap as well as subfunctionalization within the type-B *RR* gene family, the *rr21/22/23* triple mutant exhibiting decreased cytokinin sensitivity and a variety of defects in growth and development, including effects on shoot and root growth, panicle architecture, flower development, and trichome formation (Worthen et al., 2019). Here, we employ a gain-of-function approach to characterize the effects of ectopic overexpression of *RR22* in rice. To complement this gain-of-function approach, we also examined loss-of-function *Tos17* insertion alleles of *RR22*. Results from these analyses indicate that a delicate balance of cytokinin transcriptional activity is necessary for optimal growth and development in rice.

## Materials and Methods

### Plant Materials

For overexpression of *RR22*, a GFP-tagged version was driven by the *ZmUbi* promoter (Mann et al., 2012) in the pCAMBIA1300 vector (GenBank Accession Number AF234296). For this purpose, *sGFP* along with the *NOS* terminator were amplified by PCR from the plasmid pGWB5 (Nakagawa et al., 2007), and this sGFP-tNOS fragment inserted into the Pst I and Hind III sites of pCambia1300 by an In-Fusion reaction according to the manufacturer (Takara Bio United States, Inc.), creating the vector pCambia1300-MCS-GFP. The ZmUbi1 promoter was amplified from the pANIC6A vector (Mann et al., 2012), and a genomic version of *RR22* amplified from rice genomic DNA, and both fragments cloned into the Xma I and Pst I sites of the pCambia1300-MCS-GFP vector by an In-Fusion reaction. Primers used for cloning are listed in Supplementary Table 1.The resulting pZmUbi1:RR22-GFP:tNOS vector was confirmed by restriction digest and sequencing. Transformation of *Oryza sativa ssp. japonica* cv. Kitaake rice with pZmUbi1:RR22-GFP: tNOS vector in the EHA105 strain of *Agrobacterium* was performed by the Plant Transformation Facility at Iowa State University.^1^ Three RR22-OX transgenic lines, characterized by a single T-DNA insertion and high expression of the transgene, were selected for further characterization. Wild-type (WT) siblings isolated from segregating populations for each of the transgenic lines were retained as controls.

For isolation of *RR22* lines containing *Tos17* insertions, seed was obtained from the *Tos17* Rice Insertion Mutant (TRIM) collection at the National Institute of Agrobiological Sciences (NIAS) in Japan^2^ (Miyao et al., 2003; Hirochika et al., 2004). Four *Tos17* insertion alleles for *RR22* designated *rr22-1* (NF0017), *rr22-2* (ND3038), *rr22-3* (NF6804), and *rr22-4* (NG4931) were obtained for *Oryza sativa ssp. japonica* cv. Nipponbare. PCR-based screening of DNA isolated from mature leaves by the CTAB extraction method (Clarke, 2009) was used to identify homozygous *rr22* mutant lines and their WT siblings, using the primers listed in Supplementary Table 1. The locations for the *Tos17* insertions were confirmed by sequencing (Supplementary Table 2). The *rr21/22/23* CRISPR-Cas9 derived line has been previously described (Worthen et al., 2019).

### Characterization of Adult Plant Growth Parameters

Plants were grown in a greenhouse at 30°C during the day and 25°C at night, using a 10-h light/14-h dark cycle as described (Worthen et al., 2019). Panicle parameters were quantified as described, using the largest panicle from each plant characterized (Worthen et al., 2019). To visualize stigma brush hairs and trichomes, spikelets and hulls were dissected from plants right before anthesis and images captured using a Leica MZ16 microscope with a Spot Idea camera. To quantify stigma brush hair length for each line, we imaged 10 stigma brushes, with two or three stigmas isolated from each of four plants, and measured the five longest branches on each stigma brush using ImageJ. To quantify length of hull trichomes for each line, we imaged 10 hull tips, with two or three hulls imaged from each of four plants after anthesis, and measured the length of the five longest trichomes on each image.

To characterize leaf epidermal cells, flag leaves from the central tillers of mature plants were chosen, and nail polish impressions made of the abaxial side from the central region of the leaf. Approximately 1.5 cm of the leaf surface from one side of the central vein was brushed to cover with a single layer of a transparent nail polish, left on the living plant for 16 h, and then removed with a scalpel. Impressions were made of two leaves from each of five plants for each line characterized. Impressions were imaged with a Nikon 90i microscope in DIC mode and a 20x objective. Measurements of cell length and width were made with ImageJ software for 50 epidermal long cells (LC1; two different cell files from each of five specimens, with five consecutive cells analyzed per cell file; Luo et al., 2012). Cell file counts for the silica/cork cell files were performed using a Zeiss Axioscope, and all files present in the impressions were counted.

### Characterization of Seedling Growth Parameters

For growth of seedlings, seeds were sterilized and germinated as described (Worthen et al., 2019), then transferred to plastic mesh floating on Yoshida medium (Yoshida et al., 1976) supplemented with 50 μg/ml carbenicillin to control bacteria contamination in a dark plastic container. Seedlings were grown for 8 days at 30/28°C with a 12-h light/12-h dark cycle (light = 400 μE). To assess cytokinin sensitivity, the Yoshida medium was supplemented with NaOH as a vehicle control, 10 nM BA, or 100 nM BA.

### Gene Expression Analysis

For RNA sample preparation from shoots of RR22-OX lines and their corresponding WT siblings, germinated seeds were grown in sterile Kimura media as described (Worthen et al., 2019) in Solo Sundae cups (TS5R) with lids (DLR100) at 30/28°C with a 12-h light/12-h dark cycle (light = 100 μE) for 7 days. Seedlings were then removed and treated with 1 μM BA or a NaOH vehicle control for 1 h in liquid Kimura media by placing roots into the solution. Sample preparation from the *Tos17 rr22* carpels and seedlings was as described (Worthen et al., 2019). Samples were flash frozen in liquid nitrogen, ground with a tissue homogenizer (Mixer Mill 400, Retsch), and total RNA extracted using the E.Z.N.A Plant RNA Kit (Omega Bio-Tek) as described (Worthen et al., 2019). DNase treatment, first-strand cDNA synthesis, and RT-qPCR with three biological replicates, along with technical replicates, were performed as described (Worthen et al., 2019). Primers used for qRT-PCR are listed in Supplementary Table 1.

### Immunoblot Analysis

Leaf samples were flash frozen in liquid nitrogen, ground with a tissue homogenizer (Mixer Mill 400, Retsch), and total protein isolated by resuspending 50 mg of leaf powder in 500 μl of SDS-PAGE loading buffer composed of 0.12 M Tris-HCl (pH 6.8), 0.1 M EDTA, 4% (w/v) SDS, 10% (v/v) ß-mercaptoethanol, 5% (v/v) glycerol, and 0.005% (w/v) bromophenol blue. The suspension was incubated at 90°C for 10 min, centrifuged at 14,000 *g* for 2 min to pellet insoluble plant debris, proteins in the supernatant fractionated by SDS-PAGE, then electroblotted to Immobilon-P PVDF membrane (MilliporeSigma) and immundetection performed as described (Gao et al., 2008). Ponceau S staining of the nylon membrane was used to visualize proteins as a loading control (Salinovich and Montelaro, 1986). An anti-GFP antibody (B-2) from Santa Cruz Biotechnology was used as the primary antibody for detection of the RR22-GFP fusion proteins.

### Statistical Analysis

ANOVA-based analyses were performed using an online calculator.^3^ *T*-tests were performed in Excel.

## Results

### Generation of *RR22* Gain-of-Function and Loss-of-Function Lines

**Figure 1:**
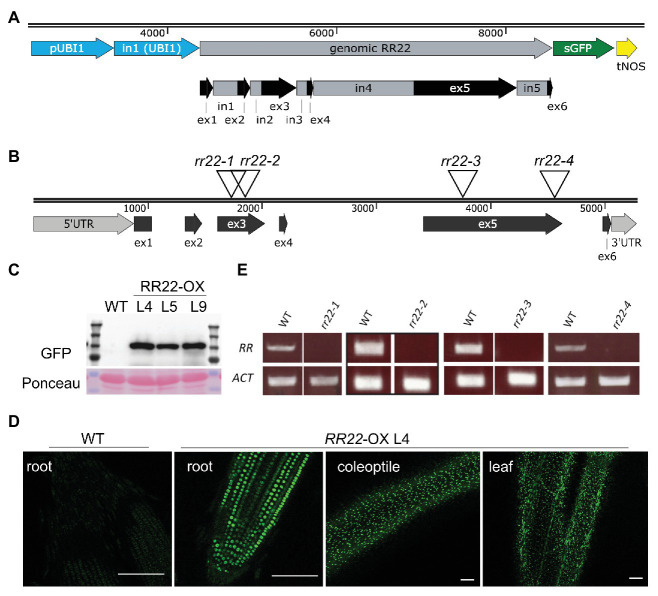
*RR22* gain-of-function and loss-of-function lines. **(A)** Structural design of the *RR22-OX* cassette. A genomic version of *RR22* was expressed from the *Zea mays UBI1* promoter incorporating the first intron of the *UBI1* gene. A sGFP fluorescent tag was encoded at the 3'-end of *RR22*. The intron/exon structure of the *RR22* gene is shown below the cassette diagram. **(B)** Insertional positions for the four *Tos17* alleles of *rr22*. **(C)** Relative protein expression levels of RR22-GFP in three independent *RR22*-OX lines as assessed by immunoblot using an anti-GFP antibody. A non-transgenic wild-type (WT) extract serves as a negative control. Ponceau S staining was used as a protein loading control. **(D)** Distribution of RR22-GFP fluorescent signal in root, dark-grown coleoptile, and leaf of *RR22*-OX L4. The WT root is a negative control. Scale bar = 100 μm. **(E)** Transcript levels of *rr22* and *ACT1* in the *Tos17 rr22* mutant lines and corresponding WT siblings assessed by semiquantitative qPCR.

To functionally characterize the role of a type-B *RR* in rice, we focused on *RR22* based on its relatively high expression level among the rice type-B *RR*s, prior indication that *RR22* plays a role in mediating cytokinin signaling, and the availability of insertion alleles through public resources (Kim et al., 2012; Tsai et al., 2012; Yamburenko et al., 2017; Worthen et al., 2019). Lines for the ectopic overexpression of *RR22* (*RR22-OX* lines) were generated by expressing a genomic copy of the *RR22* coding region from the *Zea mays UBI1* promoter. Sequence encoding a GFP tag was incorporated at the 3'-end of the *RR22* gene to allow for *in planta* visualization and assessment of relative protein levels of the *RR22-OX* transgenes (Figure 1A). Out of 15 independent transgenic lines obtained for this construct in *Oryza sativa ssp. japonica* cv. Kitaake, we selected three lines (lines L4, L5, and L9) for further characterization, based on their having a single T-DNA insertion and also exhibiting a high level of protein expression (Figure 1C). We note that the *RR22-GFP* transgenes had a tendency to decrease in expression and silence in subsequent generations, and so following their protein levels was important for maintaining ectopic overexpression in these lines. From the segregating populations, we isolated WT siblings for each of the *RR22-OX* lines to serve as controls. Based on fluorescence microscopy, RR22-GFP was expressed in root and in shoot tissues, and the protein localized in nuclei, consistent with what was observed following transient expression in rice protoplasts and with its function as a transcription factor (Figure 1D; Supplementary Figure 1A; Tsai et al., 2012).

To assist in the functional characterization of *RR22*, we obtained *Tos17* insertional mutant alleles from publicly available sources (Miyao et al., 2003; Hirochika et al., 2004; Figure 1B). The *Tos17* lines are in *Oryza sativa ssp. japonica* cv. Nipponbare, prior loss-of-function analyses having been performed through the CRISPR-Cas9 approach in *Oryza sativa ssp. japonica* cv. Kitaake (Worthen et al., 2019). Four independent *Tos17* alleles for *RR22* were brought to homozygosity from the segregating populations, and their WT siblings isolated as controls. The locations for the *Tos17* insertions were confirmed by sequencing (Figure 1B; Supplementary Table 2). All four alleles disrupt the coding region of *RR22*: the Tos17 insertions for *rr22-1* and *rr22-2* are in exon 3, and for *rr22-3* and *rr22-4* are in exon 5 (Figure 1B). None of the four *Tos17 rr22* mutants produced full-length transcripts, consistent with their being loss-of-function alleles (Figure 1E).

### Ectopic Overexpression of *RR22* Results in Cytokinin Hypersensitivity

**Figure 2:**
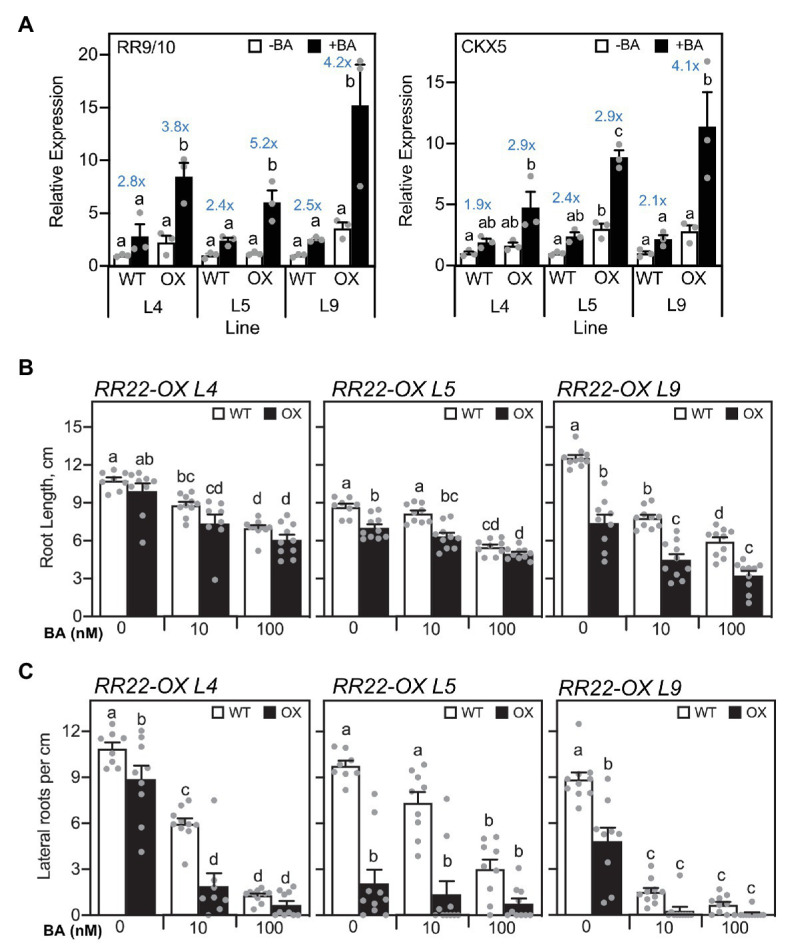
Cytokinin hypersensitive response of *RR22-OX* lines. **(A)** Expression of cytokinin-dependent genes based on qRT-PCR. Seven-day-old seedlings were treated with 1 μM BA or a NaOH vehicle control for 1 h, and gene expression examined in the shoots of the *RR22-OX* lines and their WT siblings (*n* = 3). Expression of the *ACT1* gene was used for normalization. The fold change in gene expression is indicated in blue lettering for each BA-treated to untreated sample. **(B,C)** Root growth response to cytokinin. Seminal root length **(B)** and lateral root density **(C)** were determined for 7-day-old seedlings grown on Yoshida hydroponics medium supplemented with a vehicle control, 10, or 100 nM BA (*n* ≥ 8). For statistical analyses, ANOVA with a *post hoc* Holm multiple comparison calculation was performed for each line, involving *RR22-OX* and the WT sibling of the line. Different letters indicate significant differences at *p* < 0.05.

The primary role established for type-B RRs is in mediating the transcriptional response to cytokinin (Imamura et al., 1999; Hosoda et al., 2002; Raines et al., 2016; Zubo et al., 2017). Therefore, increased expression of *RR22* is predicted to result in cytokinin hypersensitivity, which can include increased induction of cytokinin-inducible genes. To this end, we examined the expression of *RR9/10* and *CKX5*, which we previously identified as potential cytokinin primary response genes based on their rapid induction by cytokinin in multiple tissues as well as the presence of extended type-B RR binding motifs in their promoters (Raines et al., 2016; Worthen et al., 2019). We compared gene expression in shoots for the *RR22-OX* lines to their WT siblings following a 1-h treatment with 1 μM BA or a vehicle control. These treatment conditions were chosen because they result in only a modest increase in cytokinin-dependent gene expression in the WT siblings, such that the response is not saturated under these conditions (Figure 2A). All three *RR22-OX* lines exhibited enhanced induction of expression of *RR9/10* and *CKX5* in response to cytokinin as compared to their WT siblings, consistent with a heightened transcriptional response to cytokinin.

The enhanced transcriptional response of the *RR22-OX* lines to cytokinin suggests that these lines should also exhibit an enhanced response to cytokinin in growth assays. For this purpose, we examined the root growth response to cytokinin, both for seminal root length as well as for lateral root production, both of which are inhibited to various extents by cytokinin (Rani Debi et al., 2005). As shown in Figure 2B, cytokinin significantly inhibits growth of the WT seminal root, when comparing the untreated to 100 nM BA-treated roots. The roots of all three *RR22-OX* lines are shorter than their WT siblings in the absence and presence of BA, lines L5 and L9 significantly so, a result most consistent with an enhanced response to endogenous cytokinin. A more pronounced difference between the *RR22-OX* lines and their WT siblings is observed when examining lateral root production (Figure 2C). All three *RR22-OX* lines exhibit significantly reduced production of lateral roots in the absence of added BA, by 18, 78, and 45% for *RR22-OX* lines L4, L5, and L9, respectively, consistent with an enhanced response to endogenous cytokinin. In addition, lines L4 and L9 also exhibit an enhanced responsiveness to exogenous cytokinin, their production of lateral roots being reduced by 68 and 81%, respectively, when compared to their WT siblings when grown in the presence of 10 nM BA. The enhanced transcriptional response to cytokinin of the *RR22-OX* lines, based on gene expression and growth assays, is consistent with the abundance of rice type-B RRs as being rate-limiting for cytokinin signal transduction.

### Effects of *RR22* Ectopic Overexpression on Rice Growth and Development

**Figure 3:**
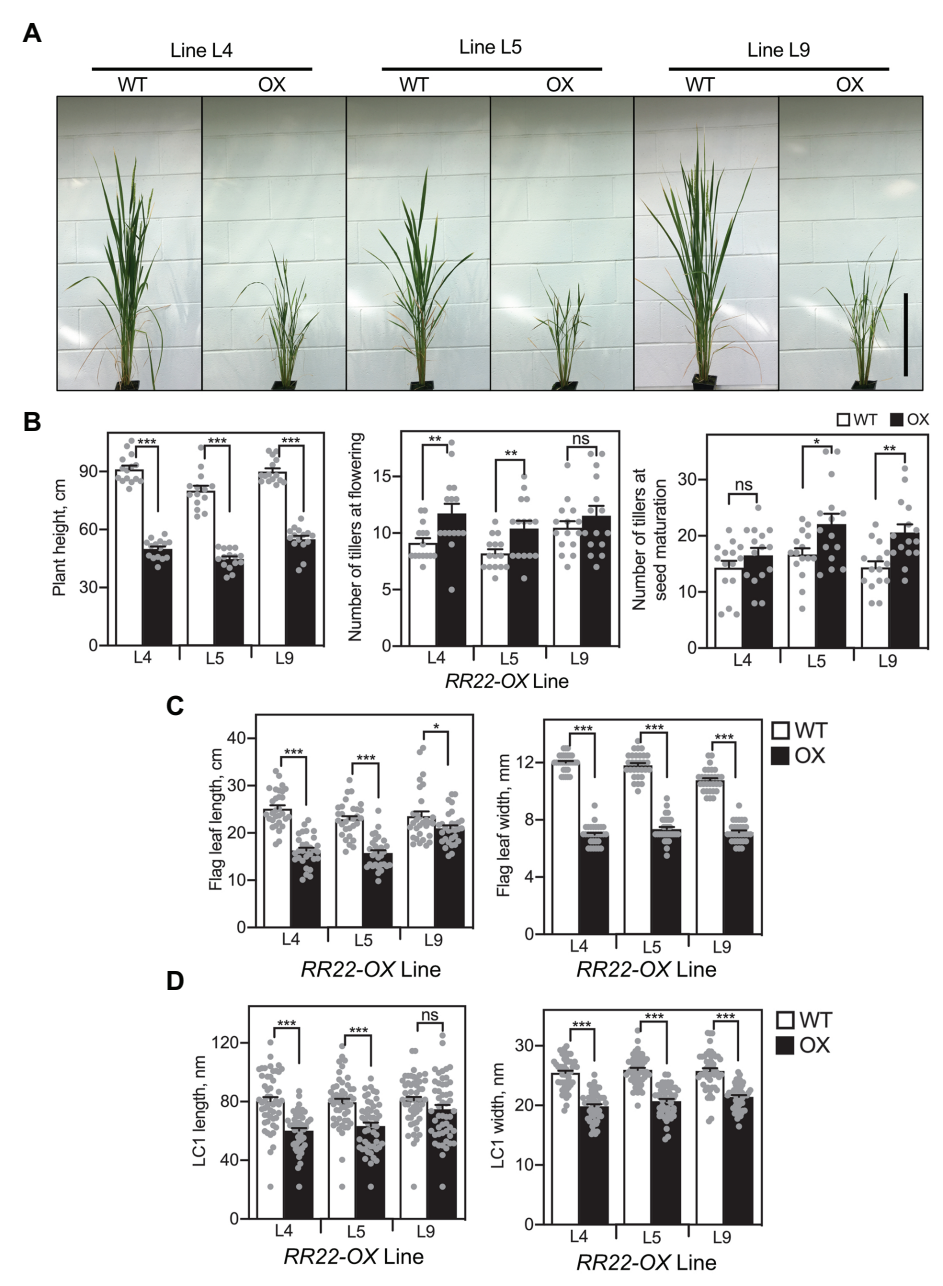
Shoot growth phenotypes of *RR22-OX* lines. **(A)** Shoots of *RR22-OX* lines and their WT siblings, taken at the initiation of flowering in the WT plants. Scale bar = 30 cm. **(B)** Plant height at flowering, number of tillers at flowering and at seed maturation. **(C)** Flag leaf length and width. **(D)** Epidermal long cell 1 (LC1) length and width of the flag leaves. Error bars show SE. The *T*-test was used for statistical comparison of each *RR22-OX* line to its WT sibling (^*^p < 0.05; ^**^p < 0.01; ^***^p < 0.001; ns, not significant).

When grown on soil, mature *RR22-OX* lines were dwarfed in comparison to their WT siblings (Figure 3). On average, the plant height for the three *RR22-OX* lines decreased 43% compared to their WT siblings. As noted earlier, the *RR22-GFP* transgene had a tendency toward silencing, and we observed a correlation between the level of RR22-GFP protein and the stature of the *RR22-OX* plants, a higher level of RR22-GFP protein being observed in the plants that exhibited the most substantial dwarfing (Supplementary Figure 1B). The *RR22-OX* lines also exhibited an elevated number of tillers (Figures 3A,B), and we observed a delay of approximately 2 weeks in flowering time compared to their WT siblings.

All three lines of the *RR22-OX* plants have shorter and narrower leaves compared to their WT siblings (Figure 3C), accounting at least in part for the reduced stature of the *RR22-OX* plants. To determine whether the decrease in leaf size of the *RR22-OX* lines was due to changes in cell proliferation and/or cell size, we examined epidermal long cells (LC1) on the abaxial surface of the flag leaf. Both length and width of the cells are reduced in the *RR22-OX* lines compared to their WT siblings (Figure 3D). The decrease in cell length (~18%) closely parallels the reduction of flag leaf length (~26%), suggesting that this decrease is primarily due to the decrease in cell length rather than any reduction in cell proliferation. Although a decrease in cell width also occurs in the *RR22-OX* lines (~20%), the decrease is not as proportionately great as that observed in flag leaf width (~38%), suggesting that other factors such as cell proliferation may also play a role in this leaf dimension.

**Figure 4:**
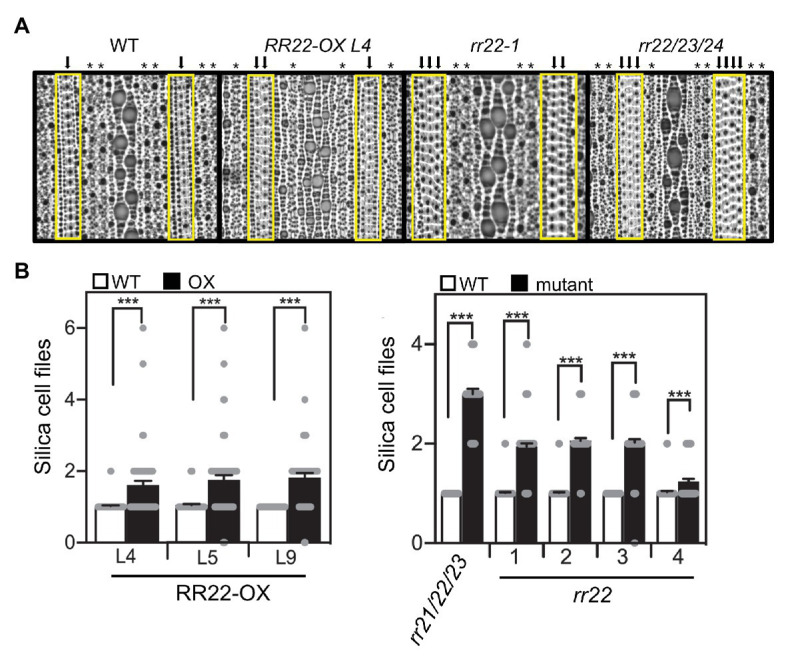
Leaf cell file phenotypes of *RR22-OX*, *rr22*, and *rr21/22/23* mutant lines. **(A)** Representative images of the abaxial epidermis of the flag leaf. The region containing the silica/cork cell files is highlighted with a yellow box, and each silica/cork cell file indicated with a black arrow. Each stomata file is indicated with an asterisk (^*^). **(B)** Quantification for the number of rows of adjacent silica/cork cell files. Impressions from five plants (two per plant) were examined and the number of rows of adjacent silica/cork cell files determined (*n* > 47). Error bars show SE. The *T*-test was used for statistical comparison of each mutant line to its WT sibling (^***^p < 0.001).

In addition to changes in cell size, we also observed effects of the *RR22-OX* lines on the development of specific cell types. The abaxial side of the rice leaf epidermis has a distinct pattern consisting of several different cell files (Luo et al., 2012). We observed effects on the number of files that contain the silica and phellem (cork) cells in the *RR22-OX* lines. In the silica/cork cell files, dumbbell-shaped silica cells alternate with cork cells and provide mechanical stability for rice leaves and improve rice resistance to pathogens (Kaufman et al., 1985). In WT plants, the silica/cork cells are typically arranged in a single file (Figure 4). However, we observed much greater variability in the number of adjacent silica/cork cell files in the *RR22-OX* lines: generally, the rows of adjacent files increased (Figure 4), although we also observed instances in which the file was no longer present (Figure 4B). In addition, we observed effects on the number of stomata files (Figure 4A; Supplementary Figure 2). In the WT flag leaves, stomata were typically arranged in two adjacent files. However, in the flag leaves of the *RR22-OX* lines, stomata were typically arranged in a single file (Figure 4A; Supplementary Figure 2), although in some cases, we also observed the sporadic appearance of stomata in a neighboring file.

**Figure 5:**
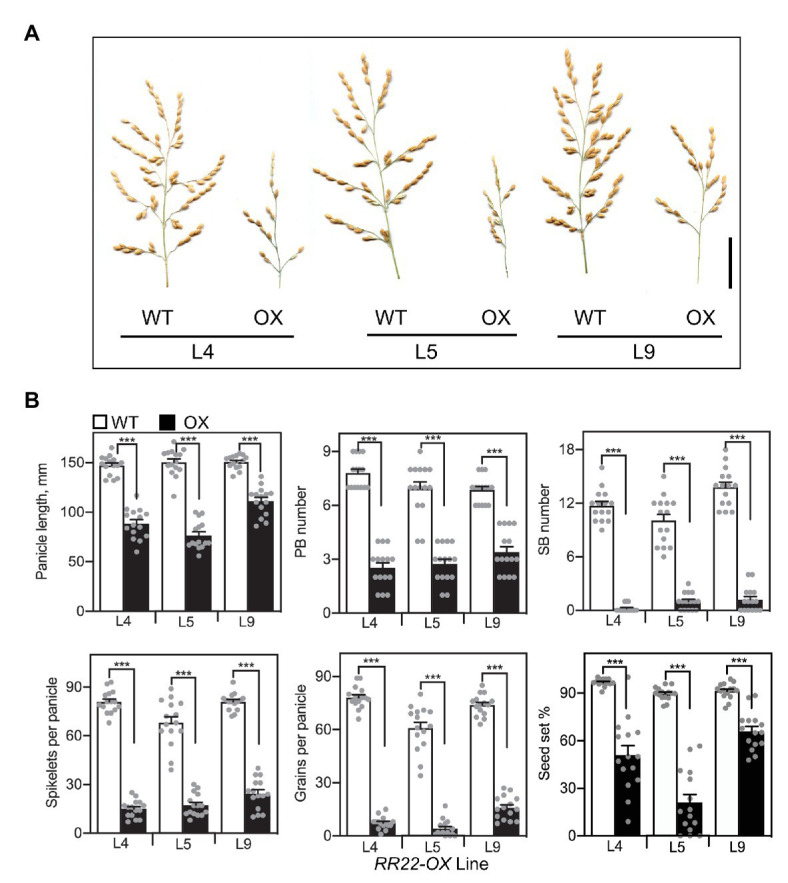
Panicle phenotypes of *RR22-OX* lines. **(A)** Panicles of *RR22-OX* lines and their WT siblings. Scale bar = 5 cm. **(B)** Yield parameters of the *RR22-OX* lines, including panicle length, primary branch (PB) and secondary branch (SB) number, spikelets and grains per panicle, and percent seed set (*n* = 15). Error bars show SE. The *T*-test was used for statistical comparison of each *RR22-OX* line to its WT sibling (^***^p < 0.001).

Overexpression of *RR22* also altered development of the rice inflorescence. Rice produces a panicle-type inflorescence, which is determinate and branched, with a defined architecture derived from the organization of the reproductive meristem (Itoh et al., 2005; Prusinkiewicz et al., 2007). The rice panicle consists of a central axis with several primary and secondary branches, each of which produces spikelets that encapsulate the rice flower. Panicles of the *RR22-OX* lines were smaller and had reduced branching compared to those of their WT siblings (Figure 5). In this respect, all three of the *RR22-OX* lines exhibited significant reductions in panicle length, primary branch number, and especially secondary branch number. The effects of the *RR22-OX* lines on panicle architecture resulted in a significant decrease in the number of spikelets per panicle (Figure 5). Seed set was also reduced in the *RR22-OX* lines and this, combined with the decreased number of spikelets, severely reduced yield based on the number of grains per panicle (Figure 5).

Cytokinin and the rice type-B *RR*s have also been implicated in trichome initiation and elongation (Maes et al., 2008; Maes and Goossens, 2010; Worthen et al., 2019). The *RR22-OX* lines produced shorter trichomes on the grain hulls than their WT siblings (Supplementary Figure 3). On the other hand, the *RR22-OX* lines had no apparent effect on the production and growth of the stigma brush hairs (Supplementary Figure 3), which are trichome-related structures involved in pollen capture whose development has been shown to be affected by cytokinin signaling (Worthen et al., 2019).

### Effects of *RR22* Loss-of-Function Mutants on Rice Growth and Development

Four *Tos17* insertion mutations of *RR22* (*rr22-1*, *rr22-2*, *rr22-3*, and *rr22-4*) were identified (Figure 1A) and characterized. These did not exhibit any prominent growth phenotypes for the shoots and roots, nor a consistent change in sensitivity for the induction of cytokinin primary response genes (Supplementary Figure 4). This is likely due to functional redundancy of *RR22* with other members of the type-B *RR* family of rice, as we previously found that the triple mutant *rr21/22/23* is of reduced stature and exhibits a reduced capacity for the induction of cytokinin primary response genes (Worthen et al., 2019). However, as described below, we were able to uncover effects of the single *rr22* mutants on the development of silica/cork files, panicle architecture, and trichomes.

As shown in Figure 4, WT leaves generally produce a single file containing alternating silica and cork cells. In contrast, the single mutants *rr22-1*, *rr22-2*, and *rr22-3* produced two adjacent files of silica/cork cells; *rr22-4* was more similar to the WT phenotype, but still produced several instances with two adjacent files of silica/cork cells. Analysis of the triple mutant *rr21/22/23* revealed an even greater increase in the rows of adjacent silica/cork files, there being three rows on average (Figure 4). Based on the number of these cell files in *rr21/22/23*, along with additional internal silica/cork cells appearing within these expanded rows, the increase in cell files appears due to increased latitudinal cell proliferation.

**Figure 6:**
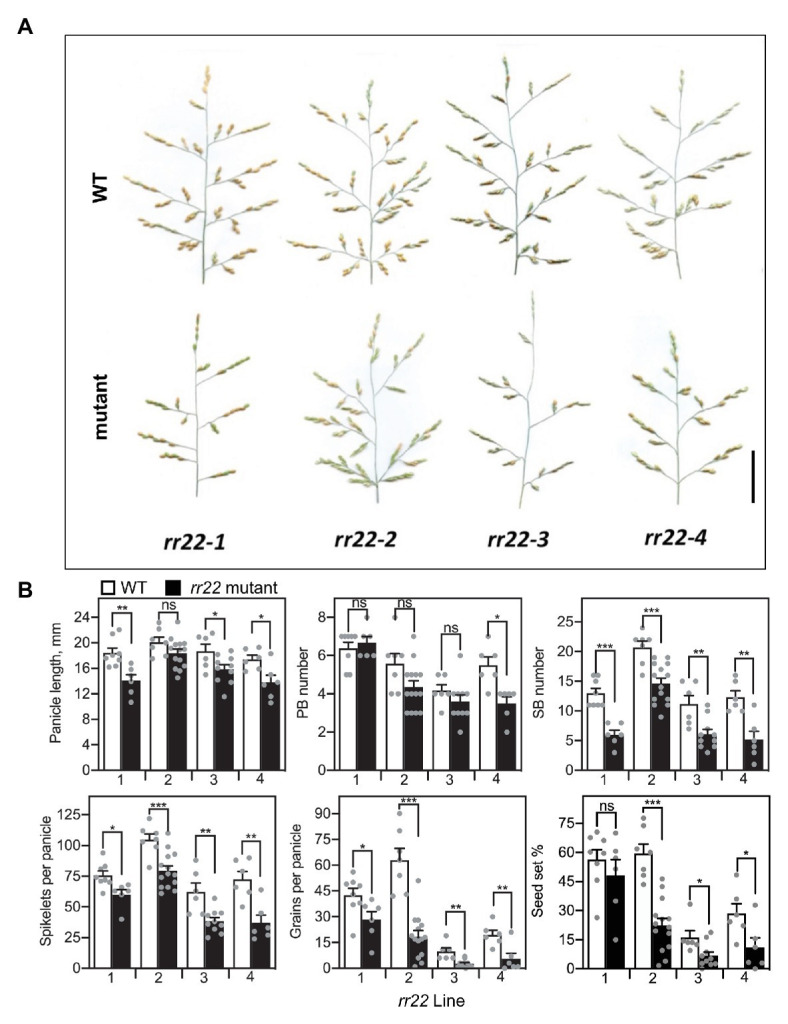
Panicle phenotypes of *Tos17 rr22* lines. **(A)** Panicles of *rr22-1*, *rr22-2*, *rr22-3*, and *rr22-4* lines and their WT siblings. Scale bar = 5 cm. **(B)** Yield parameters of the *rr22* lines, including panicle length, PB and SB number, spikelets and grains per panicle, and percent seed set (*n* ≥ 6). The *T*-test was used for statistical comparison of each *rr22* line to its WT sibling (^*^p < 0.05; ^**^p < 0.01; ^***^p < 0.001; ns, not significant).

The panicles of the *rr22* mutants were smaller than those of their WT siblings, primarily due to a reduced branching complexity (Figure 6). Analysis of various parameters associated with panicle architecture revealed that three out of four *rr22* alleles exhibited significant decreases in panicle length. The *rr22* mutants produced similar numbers of primary branches to their WT siblings but, strikingly, all four *rr22* mutant alleles exhibited a significant reduction in secondary branch number (Figure 6). These effects on panicle architecture result in a significant decrease in the total number of spikelets per panicle compared to WT. Due to fewer spikelets per panicle, coupled to a decrease in seed set, the *rr22* mutants produce fewer grains per panicle than their WT siblings (Figure 6).

**Figure 7:**
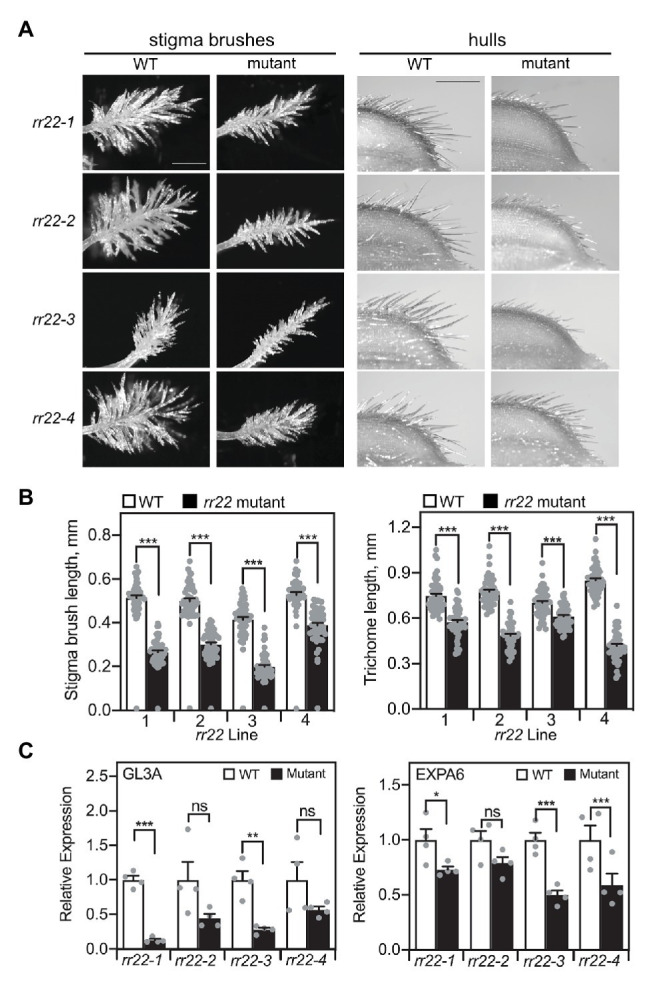
Trichome-related phenotypes of *Tos17 rr22* lines. **(A)** Stigma brushes (scale bar = 0.5 mm) and trichomes on grain hulls (scale bar = 1 mm). **(B)** Stigma brush hair length and hull trichome length, with the five longest hairs or trichomes measured per stigma or hull, respectively (*n* = 50). **(C)** Expression analysis by qRT-PCR of the stigma-enriched genes *GL3A* and *EXPA6* in carpels of *rr22* mutants and their WT siblings (*n* = 4). Expression of the *UBQ5* gene was used for normalization. Error bars are for SE. The *T*-test was used for statistical comparison of each *rr22* line to its WT sibling (^*^p < 0.05; ^**^p < 0.01; ^***^p < 0.001; ns, not significant).

The *rr22* mutants exhibited defects in the development of several types of trichomes (Figure 7). We previously found that the triple type-B *RR* mutant *rr21/22/23* was defective in the development of macro trichomes on leaves and grain hulls, as well as in the production of the stigma brush hairs (Worthen et al., 2019). We therefore examined the length of hull trichomes and stigma brush hairs in the *Tos17 rr22* lines, finding these significantly reduced in length when compared to those of their WT siblings (Figures 7A, B). Additionally, stigma-enriched genes exhibited reduced expression in carpels of the *rr22* lines when compared to their WT siblings (Figure 7C; Supplementary Figure 5). Expression of *GL3A*, which encodes a rice homolog of the transcription factor GLABRA3 of Arabidopsis, and of *EXPA6* was reduced in all four *rr22* lines (Figure 7C). Expression of two other stigma-enriched genes (*GH3.1* and *WDA1*) was less consistently reduced (Supplementary Figure 5). All four of these genes were previously found to be significantly downregulated in the *rr21/22/23* mutant, which exhibits a more severe stigma brush hair defect than the *rr22* single mutants; the more variable effects on downregulation of these genes in the *Tos17 rr22* lines are thus likely due to the intermediate mutant phenotype for the brush hairs and functional overlap with the other type-B *RR*s in the regulation of their development.

## Discussion

We recently employed a CRISPR/Cas9 gene editing approach to target the four most abundant type-B *RR*s of rice (*RR21*, *RR22*, *RR23*, and *RR24*), results from that analysis confirming a role in cytokinin signaling and revealing various roles in the growth and development (Worthen et al., 2019). As a complement to this CRISPR/Cas9-based loss-of-function approach, we have now employed a gain-of-function approach to characterize the effects of ectopic overexpression of *RR22* in rice, as well as characterized four *Tos17* loss-of-function alleles of *RR22*. Below, we discuss these results in relationship to cytokinin sensitivity, inflorescence architecture, grain yield, the production of trichome-related structures, and the growth and development of leaves and roots.

Genetic analysis of the type-B *RR*s indicates that they can be rate-limiting for cytokinin signal transduction in rice and Arabidopsis. There is functional overlap among members of the type-B *RR* family such that the overall abundance of the type-B *RR*s correlates reasonably well with the effect of loss-of-function mutations on cytokinin-dependent gene expression as well as on some physiological responses in both rice (Tsai et al., 2012; Yamburenko et al., 2017; Worthen et al., 2019) and Arabidopsis (Mason et al., 2005; Argyros et al., 2008; Hill et al., 2013). Furthermore, increased expression of *RR22* in rice, as we show here, and of the Arabidopsis type-B *RR*s *ARR1* and *ARR10* can confer cytokinin hypersensitivity on plants (Sakai et al., 2001; Zubo et al., 2017). However, although cytokinin sensitivity can be modulated by altering the expression levels of type-B RRs, the degree to which these alterations affect growth and development can vary depending on the process, tissue, and organ involved, as discussed in more detail below.

Prior studies emphasize the critical role that cytokinin levels play in regulating meristematic activity and inflorescence architecture in rice (Ashikari et al., 2005; Kurakawa et al., 2007; Gu et al., 2015; Yeh et al., 2015). Rice and Arabidopsis both produce inflorescences; however, the architecture of these inflorescences is quite different and arises from differences in the development of their inflorescence meristems (Itoh et al., 2005; Prusinkiewicz et al., 2007). Rice produces a panicle-type inflorescence, which is determinate and branched, whereas Arabidopsis produces a simple raceme-type inflorescence, which is indeterminate and unbranched. The rice panicle consists of a central stem with several primary and secondary branches, each of which produces the floral structures, called spikelets (Itoh et al., 2005). The branches are produced by the primary and secondary branch meristems and the spikelets by spikelet meristems. Disruption of the *LOG* gene, involved in cytokinin biosynthesis, produces a smaller panicle and reduced grain yield due to a failure to maintain meristematic cells in both inflorescence and spikelet meristems (Kurakawa et al., 2007; Gu et al., 2015). Of particular interest has been the discovery that a reduction in the expression of the cytokinin oxidase gene *CKX2* results in a larger panicle due to an elevation in cytokinin levels, suggesting that grain yield can be manipulated by modulating cytokinin activity (Ashikari et al., 2005; Yeh et al., 2015).

Our analyses of loss‐ and gain-of-function type-B *RR* mutants of rice emphasize the sensitivity of panicle architecture to alterations in cytokinin signal transduction. *RR21*, *RR22*, and *RR23* are all expressed during the initial stages of panicle development when primary and secondary branch meristems are produced (Yamburenko et al., 2017), and we find that the loss of a single type-B *RR* compromises the panicle architecture. This is evident in all four *Tos17 rr22* loss-of-function mutants, the most pronounced effect being a reduction in secondary branch production, a similar architectural defect also occurring in CRISPR/Cas9 generated loss-of-function mutants of *RR21* and *RR23* (Worthen et al., 2019). Interestingly, we did not observe such a reduction in panicle size for the CRISPR/Cas9 generated mutant of *RR22*, this mutant in fact exhibiting an increase in secondary branch number (Worthen et al., 2019). This phenotypic difference between the *Tos17* and CRISPR/Cas9 alleles may arise due differences in the rice varieties examined (Nipponbare for the *Tos17* lines; Kitaake for the CRISPR-Cas9 lines) and/or functional compensation of the single *rr22* mutant by another type-B *RR* family member. The latter is a possibility because the triple *rr21/22/23* CRISPR/Cas9-generated mutant exhibited substantially smaller panicles with a reduction in both primary and secondary branch production, indicating functional overlap among the type-B *RR*s in the regulation of panicle architecture (Worthen et al., 2019).

The results from *CKX2* mutants (Ashikari et al., 2005; Yeh et al., 2015), in which an increase in cytokinin levels resulted in a larger more branched panicles might suggest that increased activity of signal transduction components could phenocopy this effect. However, this was not the case based on our analysis of the *RR22-OX* lines. Instead, the *RR22-OX* lines exhibited substantially smaller panicles due to reductions in both primary branch and secondary branch production, the production of secondary branches being almost eliminated. This may suggest a heightened cytokinin response due to overexpression is inhibitory, and/or that an expanded zone of cytokinin activity due to ectopic expression results in a lack of proper spatial temporal coordination of signaling within the meristematic tissues of the panicle.

Grain yield per panicle is reduced in the *rr22* loss-of-function mutants due to a reduction in spikelet production coupled to a reduction in seed set. The reduced seed set is likely due in part to a defect in stigma brush development, because the stigma brushes of grasses play an important role in pollen capture, hydration, and guidance of the pollen tube toward the ovary (Heslop-Harrison et al., 1984; Heslop-Harrison and Reger, 1988). Each brush hair in rice is multicellular and branched, and our prior analysis indicates that type-B RR mutants severely affect cell proliferation of the brush hairs but not their cell expansion (Worthen et al., 2019). Our results from the *Tos17 rr22* mutants confirm a predominant role for *RR22* in mediating this effect on stigma brush development. The stigma brush is an epidermal trichome-related structure and we also observed that the *Tos17 rr22* mutants exhibited reduced growth of trichomes on their seed hulls, consistent with a general role for *RR22* in modulating trichome growth and development. Interestingly, the *RR22-OX* lines affected growth of the hull trichomes, but had no apparent effect on growth and development of the stigma brush structure.

Alterations in type-B *RR* activity also affect the growth and development of rice leaves. A recent study reported that the overexpression of the rice type-B RR *ORR2*, which is equivalent to *RR22* from our study (Schaller et al., 2007), reduced plant height by approximately 20% but reported no additional effects on growth and development (Shi et al., 2020). We observed a substantially greater decrease in plant height of over 40% in our overexpression lines, potentially due to higher levels of *RR22* expression in our study. The decrease in plant height of the over-expression lines, based on our study, can be largely attributed to a decrease in leaf growth, this primarily arising due to a decrease in cell size rather than cell proliferation. This effect of type-B *RR* overexpression contrasts with that found in the triple mutant *rr21/22/23* of rice, which like the *RR22-OX* lines also has shorter flag leaves, but due to a decrease in longitudinal cell proliferation rather than a decrease in cell size (Worthen et al., 2019). Interestingly, ectopic overexpression of RR22 in rice did not recapitulate what was found from the ectopic overexpression of the type-B RR *ARR10* of Arabidopsis, which induced leaf cell proliferation, although this was also sometimes accompanied by a decrease in cell size (Zubo et al., 2017).

Alterations in type-B *RR* activity affected the development of rice leaves along with affecting their growth. The architecture of grass leaves differs from that of dicots such as Arabidopsis, with various cell files serving distinct functions related to the development of diagnostic cell types (Kaufman et al., 1985; Luo et al., 2012). Both loss‐ and gain-of-function type-B *RR* mutants affected the number of silica/cork cell files, the *rr* mutants resulting in an increase in the number of adjacent files, likely due to an increase in lateral cell proliferation, whereas ectopic overexpression of *RR22* resulted in a greater variability in the number of adjacent cell files. Ectopic overexpression of *RR22* also resulted in a decrease in the number of adjacent stomatal cell files. This effect on stomata development may involve the genes *SHR* and *SCR*, because these partner transcription factors positively regulate the formation of stomatal cell files in rice (Schuler et al., 2018; Buckley et al., 2019; Wu et al., 2019) and, based on studies in Arabidopsis, cytokinin can inhibit *SCR* expression (Zhang et al., 2013). These results suggest that, in addition to affecting cell size and proliferation, that alterations in the cytokinin transcriptional response can also affect the developmental architecture of the rice leaf.

Our results are also consistent with a role for cytokinin in regulating root architecture in rice. Overexpression of *RR22* inhibited seminal root growth and had an even more pronounced effect on lateral root formation. Such an effect is consistent with cytokinin and the type-B RRs serving as negative regulators of primary root growth and lateral root development, based on studies in dicots such as Arabidopsis and tobacco as well as the monocot rice (Mason et al., 2005; Dello Ioio et al., 2007; Werner et al., 2010; Hill et al., 2013; Zubo et al., 2017). We did not note any obvious developmental abnormalities in roots of the *rr22* mutants, although *RR22* is one of the more abundantly expressed members of the type-B *RR* family in the root, shoot, and inflorescence of rice (Tsai et al., 2012; Yamburenko et al., 2017). The lack of *rr22* root phenotypes is likely due to functional overlap of *RR22* with the other type-B *RR*s based on our prior analyses of the CRISPR/Cas9-generated type-B *RR* mutants (Worthen et al., 2019), that study indicating that a triple *rr21/22/23* mutant exhibited defects in root development as well as root growth sensitivity to exogenous cytokinin. The *rr22* single mutant phenotypes we do observe in inflorescence architecture, stigma brush development, and silica/cork cell production may arise due to the sensitivity of those tissues to small changes in cytokinin activity and/or a relatively greater role for *RR22* compared to other type-B *RR*s in their developmental regulation.

In conclusion, altered expression of the type-B RRs, mediated either by loss-of-function or ectopic overexpression, results in compromised growth and development in rice. Ectopic overexpression of *RR22* in rice appears to hamper growth and development more severely than is the case when a similar study was performed in Arabidopsis (Zubo et al., 2017). The ectopic overexpression of ARR10 in Arabidopsis closely parallels expectations for increased cytokinin signaling, resulting in increased cell proliferation in the shoot and the inhibition of root growth, the opposite of what is found with loss-of-function mutants of the type-B RRs, and with no major defects in inflorescence development. In rice, on the other hand, ectopic overexpression compromises growth of the leaves and the inflorescence in a manner reminiscent to what is observed in type-B *RR* loss-of-function mutations (Worthen et al., 2019). Two possibilities, not mutually exclusive, may explain these phenotypic observations. First, that an optimal level of cytokinin activity is required to mediate these responses, with either a decrease or increase in activity compromising growth and development. Second, due to the substantial differences in inflorescence and leaf development between rice and Arabidopsis, rice is more sensitive to expanded zones of cytokinin activity arising from the ectopic expression of the type-B RR. Regardless, these studies demonstrate that a delicate balance of cytokinin transcriptional activity is necessary for optimal growth and development in rice.

## Data Availability Statement

The original contributions presented in the study are included in the article/Supplementary Material. Further inquiries can be directed to the corresponding author.

## Author Contributions

GS, JK, MY, and JW designed the research. GS and SNS provided support and oversaw the research. MY, JW, AZ, BA, and AC performed the experiments. MY, JW, AZ, BA, and SS analyzed the data. GS, MY, and JW wrote the manuscript with contributions from all authors. All authors contributed to the article and approved the submitted version.

### Conflict of Interest

The reviewer JH is currently organizing a Research Topic with one of the authors GS. The review process met the standards of a fair and objective review.

The remaining authors declare that the research was conducted in the absence of any commercial or financial relationships that could be construed as a potential conflict of interest.

## Footnotes

^1^https://www.biotech.iastate.edu/ptf/

^2^https://tos.nias.affrc.go.jp/

^3^http://astatsa.com/OneWay_Anova_with_TukeyHSD/
