# Supplementary material for: Functional Analysis of the Rice Type-B Response Regulator RR22
Source: Front Plant Sci. 2020 Nov 10;11:577676. doi: 10.3389/fpls.2020.577676 (PMC7683409; doi:10.3389/fpls.2020.577676)
Supplement: Supplementary file 1 [file Data_Sheet_1.pdf]

Supplementary Material

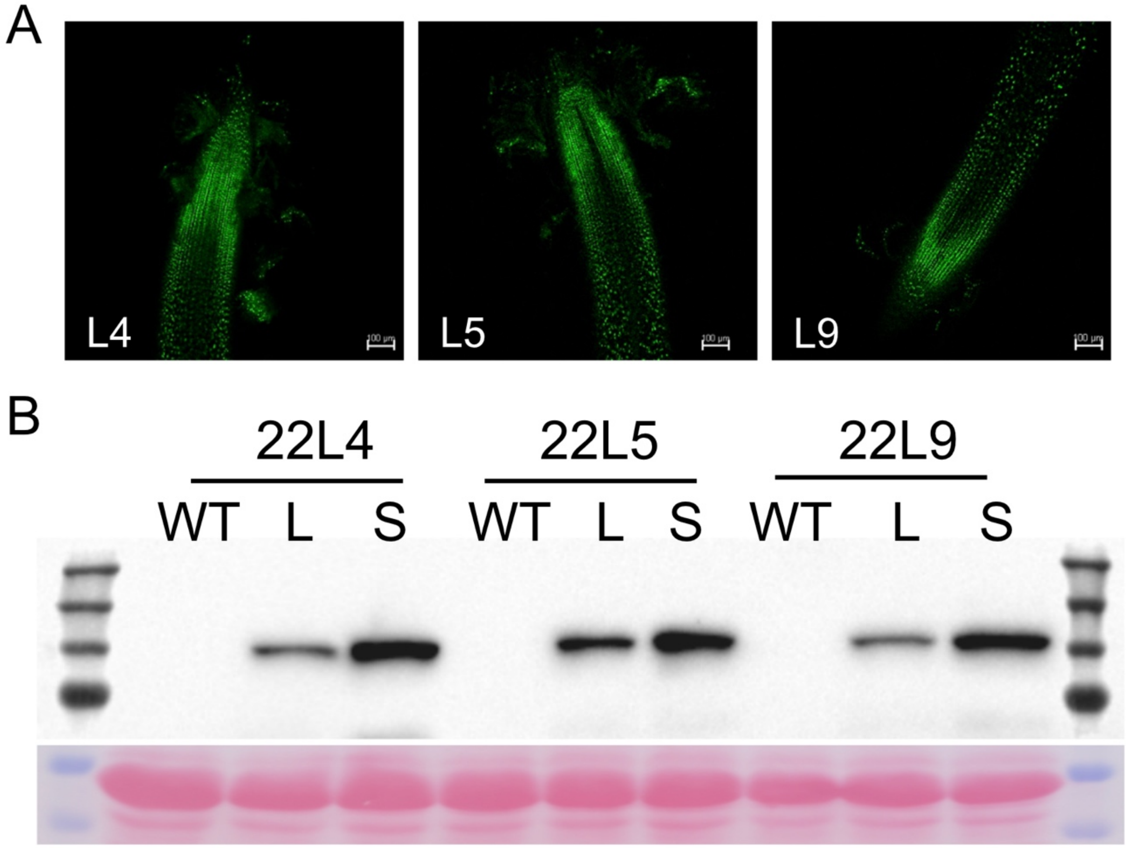

Supplementary Figure 1. Protein expression of the *RR22-OX* lines. **(A)** RR22-GFP signal in roots of seven independent *RR22-OX* lines. Seven-day-old seedlings were examined by fluorescent confocal microscopy. Scale bar=100 µm. **(B)** Protein levels of RR22-GFP in flag leaves of *RR22-OX* lines, demonstrating correlation of protein level with plant phenotype based on immunoblot analysis. The five largest plants (L), and five smallest plants (S) of 15 total were harvested from each line and subjected to immunoblot analysis with an anti-GFP antibody. Higher levels of RR22-GFP protein are found in the extracts from smaller plants compared to larger plants. Wild-type (WT) siblings serve as negative controls for RR22-GFP protein expression.

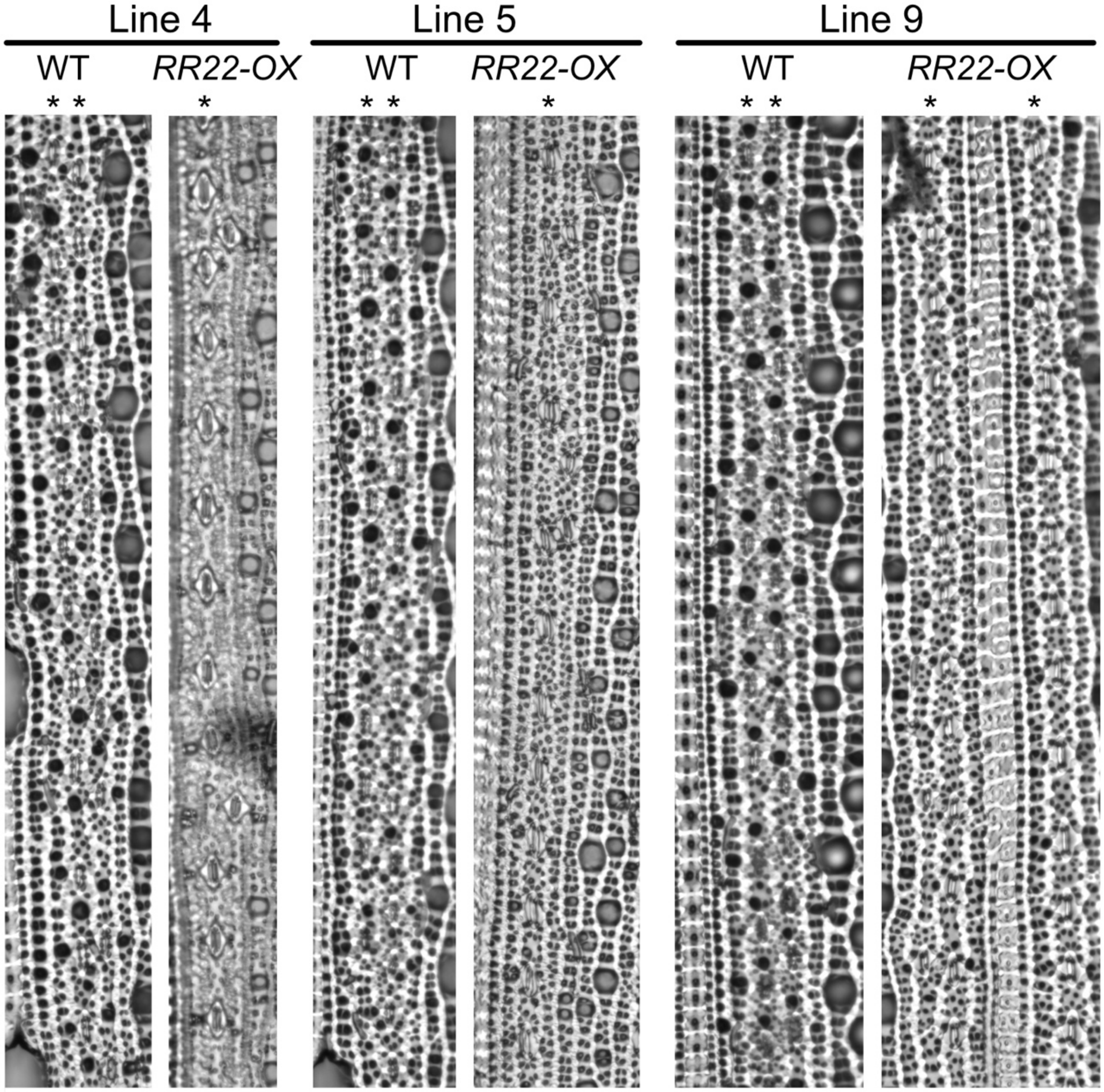

Supplementary Figure 2. Stomata phenotypes of *RR22-OX* lines. Representative images of the abaxial epidermis of the flag leaf of *RR22-OX* lines and their wild-type (WT) siblings, from independent lines 4, 5, and 9. Each stomata file is indicated with an asterisk.

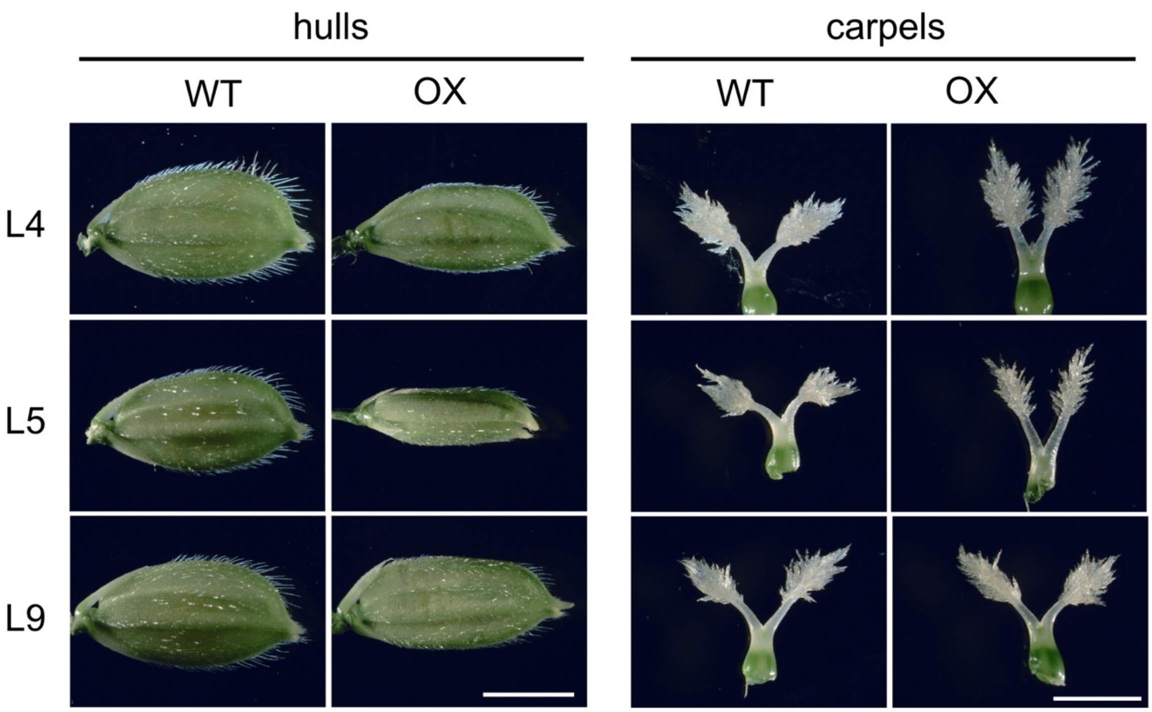

Supplementary Figure 3. Trichome-related phenotypes of *RR22-OX* lines. RR22-OX lines produce shorter trichomes on the grain hull compared to their wild-type (WT) siblings (scale bar=3 mm), although the stigma brush size of the carpels is similar to that of the wild type (scale bar=1 mm).

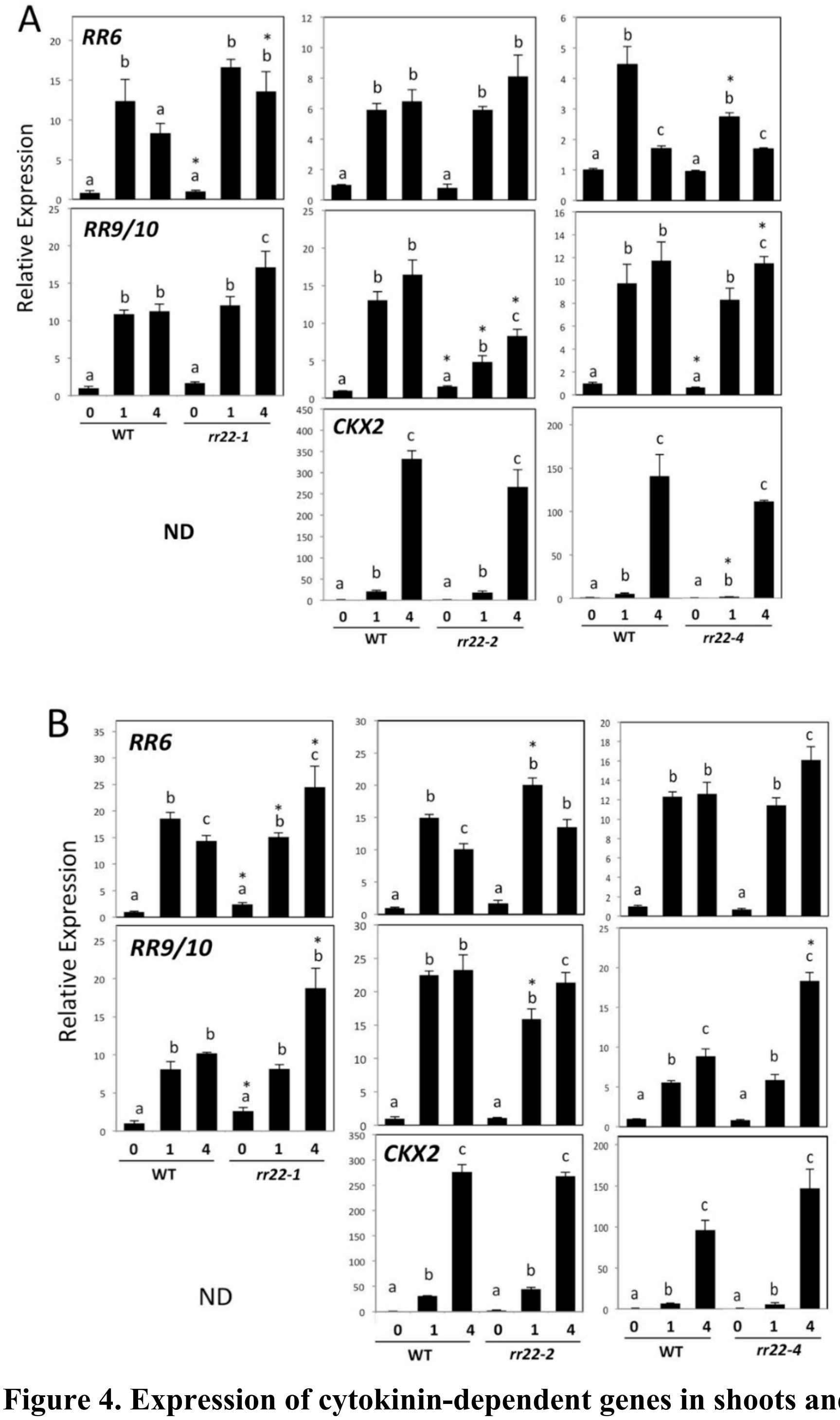

Supplementary Figure 4. Expression of cytokinin-dependent genes in shoots and roots of *Tos17 rr22* lines. Seven-day-old *rr22* seedlings and their wild-type (WT) siblings were treated with 5 µM BA for 0, 1, or 4 hr, and their roots **(A)** and shoots **(B)** harvested for analysis by qRT-PCR. Expression levels of the cytokinin-responsive genes *RR6*, *RR9/10*, and *CKX2* were determined. Expression of the *UBQ5* gene was used for normalization. ANOVA analysis was performed with post hoc Holm multiple comparison calculation within a genotype for the cytokinin response (letters), whereas a T-test is used for comparison at the same cytokinin concentration across genotypes (* $P<0.05$).

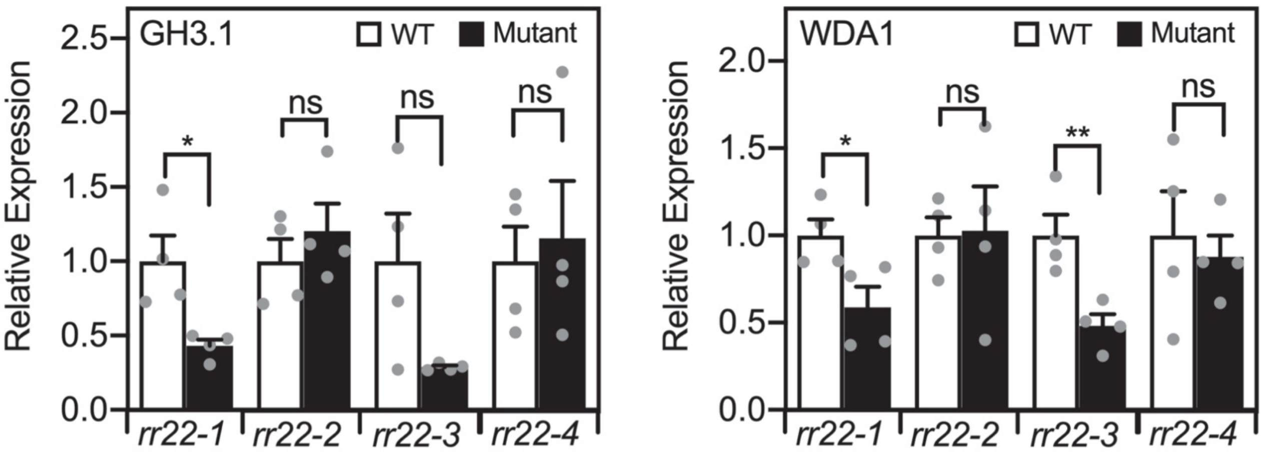

Supplementary Figure 5. Expression analysis by qRT-PCR of the stigma-enriched genes *GH3.1* and *WDA1* in carpels of *rr22* mutants and their wild-type (WT) siblings. Expression of the *UBQ5* gene was used for normalization. Error bars are for SE. The T-test was used for statistical comparison of each *rr22* line to its wild-type sibling (n = 4; * $P<0.05$; ** $P<0.01$; *** $P<0.001$; ns, not significant). *GL3A* and *EXPA6* from the same sample set are shown in **Figure 7**.

Supplementary Table 1. Primers used for this study.

**Genotyping *Tos17 rr22* and *RR-OX* lines**	
**Name**	**Sequence (5’-3’)**
Tos17-3' end	AGGTTGCAAGTTAGTTAAGA
Tos17-5' end	GAGAGCATCATCGGTTACATCTTCTC
rr22-1 (NE0017) forward	CGGTCCGAATCGAAGAACTA
rr22-1 (NE0017) reverse	CTTGCAACATTTTCCCTGGT
rr22-2 (ND3038) forward	CGTGAGCGAAACAATCTTGA
rr22-2 (ND3038) reverse	CTTGCAACATTTTCCCTGGT
rr22-3 (NF6804), *rr22-4* (NG4931) forward	GCTTGGAAGAAAACCAGCAG
rr22-3 (NF6804), *rr22-4* (NG4931) reverse	CAGTTTTGGCATTTTTGGCT
HygroR-Forward	GCCGCGCTCCCGATTCCGGA
HygroR-Reverse	GCTCGAAGTAGCGCGTCTGCT
**Generation of pZmUbi1:RR22-GFP:tNOS vector**	
sGFP-tNOS forward	cctctagagtcgacctgcagATGGTGAGCAAGGGCGAG
sGFP-tNOS reverse	ggccagtgccaagcttGATCTAGTAACATAGATGACACCGCG
pZmUbi1 forward	gagctcggtacccgggTGCAGTGCAGCGTGACCCGG
pZmUbi1 reverse	TGCAGAAGTAACACCAAACAACAGGGTGAG
OsRR22 forward	ggtgttacttctgcaATGCTTCTGGGTGCTTTGAGGATG
OsRR22 reverse	cccttgctcaccatctgcagTATGCAGGCACCAAGTGGAAAAAGG
**Semi-quantitative PCR**	
OsRR22 (+108) F	GTCGACGATGACCCGGTG
OsRR22 (1996) R	GGCCATCAAAATTGCAGCCA
**Quantitative real time PCR**	
RR6-Forward	CATCACCGACTACTGGATGC
RR6-Reverse	GGGATCTCCTTGAGCTGAGA
RR9/10-Forward	TCATGAGGACAGCCCAATTTCTA
RR9/10-Reverse	TGCAGTAGTCTGTGATGATCAGGTT
CKX5-Forward	CCCCATGAACAGGCACAAGT
CKX5-Reverse	GAGGATCTCCCGGTTCTGCC
CKX2-Forward	GTGGCCGGGATAGCCTAC
CKX2-Reverse	AGTGCCGCTTCTGCCACT
ACT1-Forward	GGTATTGTGTTGGACTCTGG
ACT1-Reverse	CCGTTGTGGTGAATGAGTAA
UBQ5-Forward	ACCACTTCGACCGCCACTACT
UBQ5-Reverse	ACGCCTAAGCCTGCTGGTT
GL3A-Forward	CCAGCAGCAGCGAAAAATACA
GL3A-Reverse	GAAGCCGTCCTTCCAAGTCA
EXPA6-Forward	AAATCCCTACGCAGTGCCAG
EXPA6-Reverse	TTGGGGTAGTTTGGTGCGTT
GH3.1-Forward	CTCCATACCGCTGCCTACAT
GH3.1-Reverse	TTGTGCTCCCTGGATTTCGG
WDA1-Forward	AACACTTACCGCTGTGGAGG

WDA1-Reverse	TAGGCAACGAGCTCAGCAAA

Supplementary Table 2. Border junctions for *Tos17 rr* mutant lines.

Line	Location of insert	GENE/non-gene junction (5’ - 3’)
rr22-1	Exon 3 (+ 858)	CATGTTGTTAGGAtcttaactaacttgc
rr22-2	Exon 3 (+ 978)	TTCTGATCAAAATGGtcttaactaacttgc
rr22-3	Exon 5 (+ 2892)	AACAGAACTGGATTCtcttaactaacttgc
rr22-4	Exon 5 (+ 3577)	CTGCAGCTTTGAACTtcttaactaacttgc
